# Refractory Immune Thrombocytopenic Purpura With Low Immature Platelet Fraction

**DOI:** 10.7759/cureus.82533

**Published:** 2025-04-18

**Authors:** Kabeer Ali, Tiffany Scotto, Jeremy M Williams, Pramod Reddy

**Affiliations:** 1 Internal Medicine, University of Florida College of Medicine – Jacksonville, Jacksonville, USA

**Keywords:** immature platelet fraction, immune mediated thrombocytopenic purpura (itp), intravenous immunoglobulin (ivig), ipf, refractory itp, refractory itp management, steroid refractory itp

## Abstract

Diagnosis of immune thrombocytopenic purpura (ITP) remains challenging due to the lack of a "gold standard test," with current approaches relying on clinical evaluation, complete blood count, and peripheral blood smear. The immature platelet fraction (IPF) has emerged as a useful tool for differentiating ITP from hypoproliferative thrombocytopenia, typically presenting as elevated in ITP cases. IPF measures the percentage of immature platelets and is primarily used to help differentiate between thrombocytopenia caused by decreased production, such as bone marrow disorders, and destruction, like ITP.

We present a unique case of a 43-year-old woman with severe thrombocytopenia, initially presenting with a platelet count of zero and a low IPF. Despite multiple platelet transfusions and high-dose dexamethasone, her platelet count remained critically low. Bone marrow biopsy findings confirmed ITP, leading to a revised treatment strategy that included intravenous immunoglobulin (IVIG) and rituximab, resulting in sustained platelet recovery. This case underscores the diagnostic and therapeutic challenges associated with ITP, particularly in patients with atypical presentations. The presence of a low IPF complicated initial diagnostic considerations, necessitating a bone marrow biopsy to exclude alternative etiologies such as bone marrow failure. Additionally, patient-specific factors, including diabetes mellitus, chronic pancreatitis, and prior alcohol use disorder, may have contributed to the severity of thrombocytopenia. Our findings highlight the need for individualized diagnostic approaches and tailored treatment strategies in severe and refractory ITP cases.

## Introduction

Immune thrombocytopenic purpura (ITP) is an autoimmune disorder characterized by isolated thrombocytopenia due to increased peripheral platelet destruction. It is due to autoantibodies targeting platelet surface antigens, resulting in rapid clearance by the reticuloendothelial system. Its presentation is heterogeneous, and a patient can range from being asymptomatic to requiring intensive care unit (ICU) level of care if the platelet count falls below 20 000 per microliter of blood (20 x 10^3/uL) or has symptoms of bleeding [1]. In addition to a variable presentation, its diagnosis can be challenging, with no “gold standard test” available to diagnose this condition. Current guidance relies mainly on a thorough history, isolated thrombocytopenia on complete blood count, and peripheral blood smear evaluation to exclude pseudo thrombocytopenia [2]. The immature platelet fraction (IPF) has emerged as a valuable tool for distinguishing IPF from hypo-proliferative thrombocytopenia. One study found that an IPF cut-off point of 6.3% had a sensitivity of 92.7% and a specificity of 92.5% for diagnosing ITP [3]. We highlight an atypical presentation of ITP, characterized by an initial platelet count of zero and a low IPF, that was refractory to corticosteroid therapy.

## Case presentation

**Figure 1:**
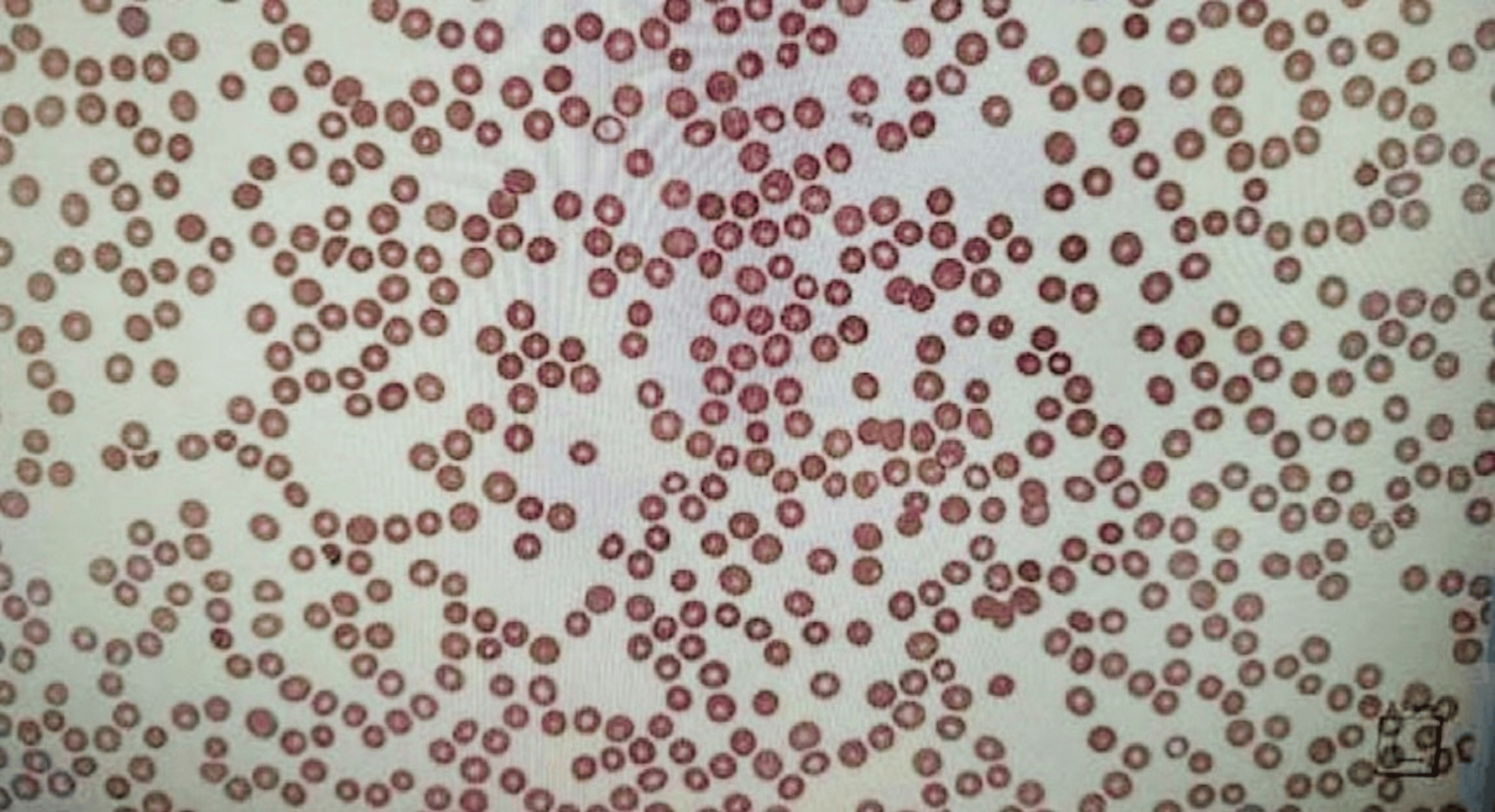
Patient's peripheral blood smear reflecting true thrombocytopenia

A 43-year-old woman with a history of diabetes mellitus (DM), chronic pancreatitis, and prior alcohol use disorder presented to the emergency department with complaints of increased bruising and vaginal bleeding that started two days prior to admission. She denied any recent trauma, antiplatelet, anticoagulation, or non-steroidal anti-inflammatory (NSAID) use, or other inciting events before symptom onset. Vital signs on presentation were significant for hypertension with a blood pressure of 155/84 millimeters of mercury (mmHg) and tachycardia to 109 beats per minute (bpm. The patient’s physical examination was notable for a large left hip hematoma with overlying ecchymosis, scattered petechiae across the chest and bilateral upper extremities, and inferior tongue bullae. The patient’s initial laboratory revealed a platelet count of 0 (reference range: 140-440 x 10E^3 per µL), hemoglobin of 9.0 grams per deciliter (g/dL) (reference range: 12-16 g/dL), and mildly elevated liver enzymes (Table 1). Peripheral blood smear showed a true thrombocytopenia without platelet clumping (Figure 1). Further workup of her anemia and thrombocytopenia revealed a normal coagulation profile, no evidence of hemolysis, and an immature platelet fraction of 0.7% (reference range: 1-7%). An infectious workup, including blood and urine cultures, was negative. Fecal occult blood test (FOBT) was negative, and pelvic ultrasound did not show any uterine lesions. Urine analysis was positive for blood and red blood cells, however it is likely that the urine sample was contaminated in the setting of vaginal bleeding. Hematology/oncology was consulted with concerns about aplastic anemia, given the low IPF value versus ITP. A trial of corticosteroid therapy with intravenous dexamethasone 40 milligrams (mg) for five days was initially recommended.

**Table 1:** Patient's laboratory investigations at presentation with reference ranges for comparison

Laboratory investigation	Patient value	Reference range
White blood cell	10.2 x 10^3 cells/µL	4-11 x 10^3 cells/µL
Hemoglobin	9.0 g/dL	11.6-15 g/dL
Platelet	0 x 10^3 cells/µL	150-400 x 10^3 cells/µL
Immature platelet fraction	0.7%	1-7%
Blood urea nitrogen (BUN)	9 mg/dL	6-20 mg/dL
Creatinine	0.6 mg/dL	0.5- 1.1 mg/dL
Lactate dehydrogenase (LDH)	202 U/L	135-214 U/L
Haptoglobin	78 mg/dL	0-200 mg/dL
Corrected reticulocyte count percentage	38 mg/dL	15-60 mg/dL
Prothrombin time (PT)	2.5%	0.5-2.5%
Activated partial thromboplastin time (aPTT)	25 seconds	25-35 seconds
International normalized ratio (INR)	1.2	0.8-1.1

**Figure 2:**
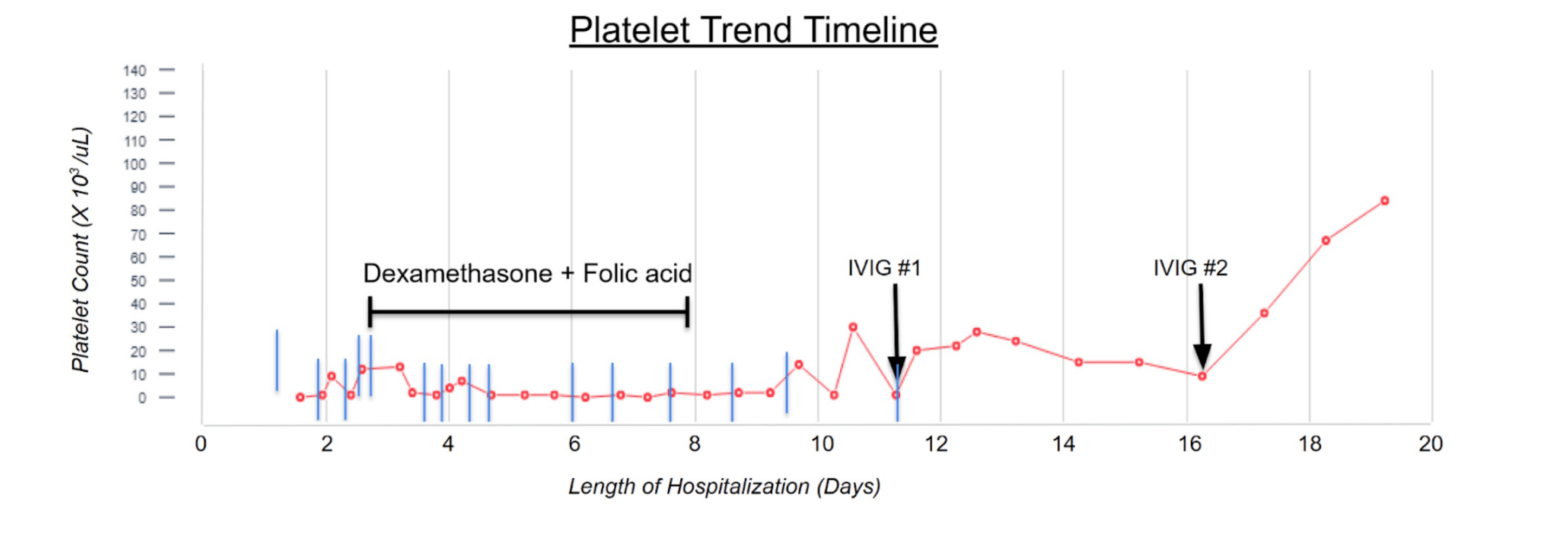
Line graph demonstrating patient's platelet trend with different interventions

Over the following 11 days, the patient received a transfusion of a total of 15 units of apheresis platelets and was given five intravenous doses of 40 milligrams (mg) dexamethasone daily and 5mg oral folic acid. However, her platelet count failed to respond appropriately, never rising above 10 x 10^3 per uL. A basic autoimmune workup revealed negative anti-nuclear antibodies (ANA) and negative antiphospholipid antibodies. A bone marrow biopsy was then performed for further evaluation, which showed increased megakaryocytes suggestive of ITP without overt evidence of leukemia, lymphoma, or myelodysplasia. The patient was then trialed with intravenous immunoglobulin (IVIG). A single dose of IVIG was given with mild improvement in platelet count to 36 x 10^3/uL; however, counts quickly returned to less than 10 x 10^3/uL in three days. An additional dose of IVIG was then given along with intravenous rituximab, which resulted in a significant improvement in thrombocytopenia to greater than 30 x 10E^/uL. Platelet counts stabilized and remained above 50 x 10^3/uL without recurrence for the remainder of her hospitalization (Figure 2). The patient was discharged on an oral prednisone taper and scheduled to receive outpatient rituximab infusions with hematology.

## Discussion

This case highlights how challenging the diagnosis and management of a patient with thrombocytopenia can be. Thrombocytopenia, as described above, is characterized by a platelet count of less than 150 × 10^3 per µL. A large percentage of hospitalized patients, especially those in the intensive care unit (ICU) setting, develop thrombocytopenia. The incidence of thrombocytopenia ranges from 8-67% [4]. This contrasts with thrombocytopenia in the outpatient setting, which is rarer and less documented. It is often detected incidentally during routine laboratory work, and often, pseudothrombocytopenia is at play [5]. 

The unique aspect of this case is not only the co-occurrence of ITP with a low IPF but also the severity of the patient’s thrombocytopenia at presentation. This patient presented with a platelet count of zero cells per microliter, which is exceptional. Platelet counts of zero are not commonly reported, even in ICU patients, and as such, not much guidance exists on managing these patients. This suggests one of two broad differentials: bone marrow failure, which can be from aplastic anemia or myelodysplastic syndrome [6]. The other possibility is rapid peripheral destruction of platelets, which can be from severe ITP, thrombotic microangiopathy (TMA), or disseminated intravascular coagulation (DIC). It constitutes a hematological emergency regardless of etiology and warrants an urgent hematology consultation. 

Outside ITP being the main driver for this patient’s severe thrombocytopenia, there were several other patient-specific factors. This patient had diabetes mellitus, and several studies have explored the association between diabetes mellitus and thrombocytopenia. One study noted a significantly lower platelet count in patients with diabetes mellitus than in non-diabetic controls [7]. Another study sought to elucidate the etiology behind this and identified the presence of anti-thrombopoietin antibodies in a subset of patients with type 2 DM [8]. There is also a link between chronic pancreatitis and thrombocytopenia, though this association is not as robust [9]. Literature suggests that patients with chronic pancreatitis are in a chronic systemic inflammatory state, which can precipitate endothelial injury and put patients more at risk for developing TMA [9]. In the same vein as chronic pancreatitis, a history of alcohol use disorder can put patients at increased risk for thrombocytopenia. However, alcohol abuse also independently causes thrombocytopenia by suppressing bone marrow function and inducing platelet apoptosis [10]. 

Historically, ITP has been challenging to diagnose, as it has no strict diagnostic criteria. It is typically defined as a platelet count of below 100,000 per cubic millimeter in patients with other causes of thrombocytopenia, which have been excluded [11]. In 2023, Kashiwaga et al. proposed diagnostic criteria for ITP involving isolated thrombocytopenia with no evidence of dysplasia of any blood cell type on the blood smear, a normal or slightly increased thrombopoietin (TPO) level, elevated IPF, and absence of other causes [12]. Using these criteria, the presence of all 4 indicates ITP, and the unavailability of TPO or IPF indicates possible ITP. The lack of measurement of TPO meant our patient had possible ITP. However, their clinical course led to a more definite diagnosis. The American Society of Hematology does not routinely recommend a bone marrow evaluation for suspected ITP, except when there are atypical features that suggest an alternative diagnosis [13]. In our case, the low IPF provided a diagnostic dilemma that led to a bone marrow biopsy being performed. Some studies have noted the incidence of a low absolute IPF (A-IPF) in ITP patients with enhanced complement activation capacity (CAC) [14,15]. Furthermore, the overlap of CAC and ITP has been reported to be as high as 59%, which suggests that the incidence of low IPF with ITP may not be as low as is currently believed [14].

## Conclusions

This case highlights the complexities of diagnosing and managing severe thrombocytopenia, particularly in ITP with an atypical presentation. Beyond the diagnostic challenges, the evolving nature of ITP and its diagnostic criteria, including the role of IPF, reinforces the need for a comprehensive approach to evaluation. This case also emphasizes the importance of early hematology consultation and individualized treatment strategies in cases of refractory ITP. Future research should explore the implications of low IPF in ITP diagnosis and its potential impact on treatment response.
